# Multivariate ordered logistic regression analysis of the postoperative effect of symptomatic discoid lateral meniscus

**DOI:** 10.1007/s00402-021-03821-3

**Published:** 2021-02-22

**Authors:** Shun-Jie Yang, Jian Li, Yang Xue, Zhong Zhang, Gang Chen

**Affiliations:** Department of Orthopedic Surgery, West China Hospital, Sichuan University, No.37, Guoxue Alley, Chengdu, 610041 China

**Keywords:** Discoid lateral meniscus, Postoperative effect, Multivariate analysis

## Abstract

**Introduction:**

The postoperative effect of arthroscopy in the treatment of symptomatic discoid lateral meniscus (DLM) varies greatly among individuals. Therefore, this study aims to investigate the factors affecting the postoperative outcomes of symptomatic DLM.

**Materials and methods:**

According to the inclusion and exclusion criteria, patients with symptomatic single-knee DLM who underwent arthroscopic surgery at our hospital from 9/2008 to 9/2015 were included. Retrospectively collected 16 factors probably affecting postoperative outcomes. The Ikeuchi grade system was used to evaluate the knee joint function. Univariate analysis was performed by Kruskal–Wallis rank-sum test or Mann–Whitney *U* test, and multivariate analysis by ordered logistic regression. *P* < 0.05 was considered statistically significant.

**Results:**

A sum of 502 patients was included, including 353 females (70.3%) and 149 males (29.7%). Difference between preoperative and postoperative Ikeuchi grade was statistically significant (*P* < 0.001). Female was bad to obtain a good Ikeuchi grade (*P* = 0.009, OR 0.458). Outerbridge grade (*P* = 0.018, OR 0.638) was negatively correlated with Ikeuchi grade. BMI (*P* = 0.001, OR 0.875) and work intensity (*P* = 0.020, OR 0.611) were inversely correlated with Ikeuchi grade. Age of onset (*P* < 0.001, OR 0.956) and symptoms duration (*P* < 0.001, OR 0.988) were negatively correlated with Ikeuchi grade. Besides, compared to total meniscectomy, meniscoplasty with a repair was an unfavourable factor for Ikeuchi grade (*P* = 0.044, OR 0.245).

**Conclusions:**

With the increase of BMI, work intensity, age of onset, duration of symptoms, and the severity of cartilage lesion, the postoperative results become worse. Moreover, female and meniscoplasty with repair are risk factors for the postoperative outcomes.

**Supplementary Information:**

The online version contains supplementary material available at 10.1007/s00402-021-03821-3.

## Introduction

The discoid meniscus has an abnormal shape and structure. The disintegration of the circular collagen fibre system in the discoid meniscus matrix may be responsible for the higher tear rate and higher degeneration of the discoid meniscus compared to the normal meniscus [1]. Clinically, compared with the discoid medial meniscus, discoid lateral meniscus (DLM) is the most common, with a high prevalence in Asian populations (16–20%) [2, 3]; and bilateral DLM accounts for 79–97% [4]. Symptomatic DLM is characterized by pain, swelling, snapping, locking, knee instability and limited mobility [5], which is mainly diagnosed by magnetic resonance imaging (MRI) and treated by arthroscopic surgery [3, 6]. Although the overall postoperative outcomes of symptomatic DLM are acceptable [1, 5, 7, 8], the outcomes between individuals are still largely different, which may result from diversity in patient’s characteristics and treatments [6]. Currently, studies have reported the influencing factors of postoperative arthroscopy for symptomatic DLM, but the results are inconsistent possibly resulted from differences in sample size, studied factors, and knee function evaluation system, etc. [9–11]. Therefore, the present research aims to investigate the influential factors of arthroscopic treatment for symptomatic DLM by retrospectively analysing more samples and factors and evaluating the knee function with Ikeuchi grade system that is simple and practical, hoping to provide theoretical basis and clinical guidance for individualized treatment and prediction of postoperative efficacy. The hypothesis is that individual variations and inconsistent treatments of different patients, such as sex, body mass index (BMI), surgical mode, etc., would influence the outcomes of arthroscopic surgery for symptomatic DLM.

## Materials and methods

**Fig. 1:**
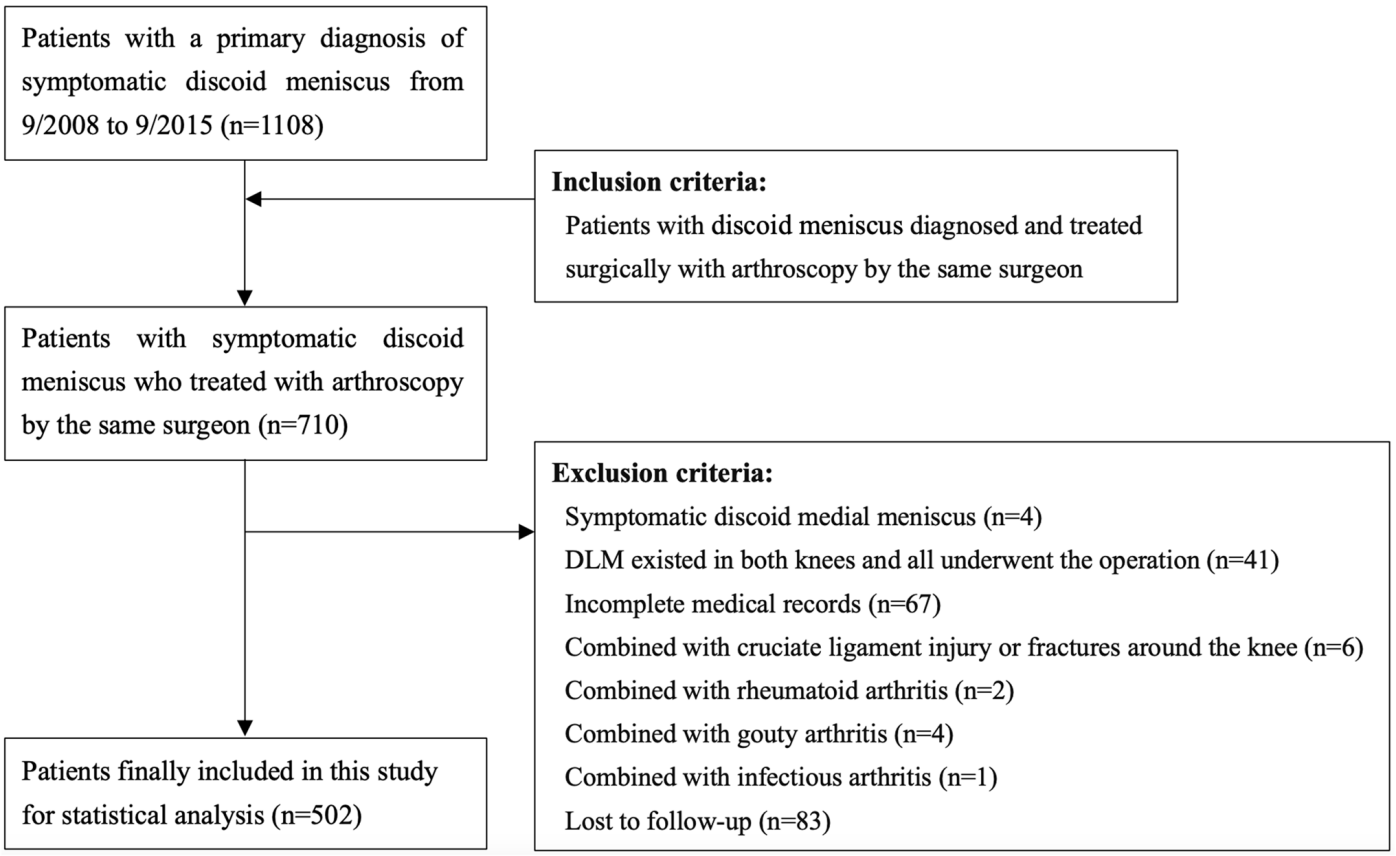
Flow chart of the study

According to the inclusion and exclusion criteria, patients with symptomatic single-knee DLM who underwent arthroscopic surgery at our hospital from 9/2008 to 9/2015 were included. The inclusion criteria, exclusion criteria and flow chart of the study were detailly shown in Fig. 1.

Patients with third-degree DLM lesion classified by Stoller MRI classification [6] and with symptoms such as pain, lock-up, and flexion and extension disorders need arthroscopic surgery. Method of arthroscopic surgery comprises meniscoplasty, meniscoplasty with repair, and total meniscectomy. Meniscoplasty is applied to the patients with continuous circular fibers in DLM, in which the broken part of the white area or red-white area in the free edge of the meniscus is removed, and about a width of 6–8 mm discoid meniscus is retained with a smooth and crescent shape. Meniscoplasty with repair is suitable for the patients with continuous circular fibers in DLM and accompanied by instability or longitudinal tear (< 10 mm) of the synovial margin, in which the torn DLM in synovial margin is repaired by total intraarticular suture method based on the meniscoplasty. Total meniscectomy is appropriate for the patients with discontinuous circular fibers in DLM (e.g. radial tear from synovial margin to free edge), in which the damaged meniscus tissue is removed as the posterior root, body, and anterior root of the meniscus tissue are not continuous, and some unfunctional residual meniscus tissue is retained. The surgical procedures for all patients were performed by the same senior surgeon whose surgical technique was reliable and stable.

The following 16 factors potentially affecting postoperative efficacy were collected from the medical records including imaging data (knee X-ray and MRI), patient history and arthroscopic surgery videos: sex; BMI; work intensity (according to the REFA daily life and work intensity classification [12], Schedule 1); trauma history (DLM lesion because of sports, falls, sprains, etc.); age of onset (years); symptoms duration (months); involved knee joint (left or right); K–L grade (according to the Kellgren–Lawrence Classification of Osteoarthritis [13], Schedule 2); DLM type (according to the Watanabe classification [9], Schedule 3); site of the meniscus tear; type of DLM tear (according to meniscus tear O'Connor classification [14], Schedule 4); combined medial meniscus tear; site of the articular cartilage lesion [including the lateral compartment (lateral femoral condyle and lateral tibial plateau), medial compartment (medial femoral condyle and medial tibial plateau), and patellofemoral joint (patella and trochlea)] [8]; severity of cartilage lesion (according to the Outerbridge grade [15], Schedule 5); mode of arthroscopic surgery (including meniscoplasty, meniscoplasty with repair, and total meniscectomy) and final follow-up time (months). Among the above factors, the intra-articular lesions such as DLM type, DLM tear, cartilage lesion, etc. were judged by arthroscopy. Postoperative efficacy was evaluated by Ikeuchi grade [16], (Schedule 6), the score of which was obtained through regular follow-up by the outpatient visit. The basic characteristics of the included subjects were shown in Table 1. The assignment of 16 factors to be studied and Ikeuchi grade were displayed in Table 2.

**Table 1:** Basic characteristics of the included patients

Demographic details	*N* (%)/*M* (range; IQR)	Demographic details	*N* (%)/*M* (range; IQR)	Demographic details	*N* (%)/*M* (range; IQR)
Patients	502	No tear	38 (7.6%)	Level I	29 (5.8%)
Sex		Anterior horn	27 (5.4%)	Level II	52 (10.4%)
Female	353 (70.3%)	Posterior horn	41 (8.2%)	Level III	23 (4.6%)
Male	149 (29.7%)	Body	204 (40.6%)	Level IV	40 (8.0%)
BMI (kg/m^2^)	22.0 (13.8 ~ 44.4; 4.3)	Two or more locations	192 (38.2%)	K–L grade	
Work intensity		O'Connor type		Level 0	375 (74.7%)
Grade 0	27 (5.6%)	No tear	38 (7.6%)	Level I	56 (11.2%)
Grade 1	212 (42.2%)	Longitudinal (/bucket handle) tear	171 (34.1%)	Level II	44 (8.8%)
Grade 2	240 (47.8%)	Horizontal tear	154 (30.7%)	Level III	23 (4.6%)
Grade 3	22 (4.4%)	Oblique tear	10 (2.0%)	Level IV	4 (0.8%)
Grade 4	1 (0.2%)	Transverse (/radial) tear	45 (9.0%)	Surgical method	
Trauma history		Variant tear	84 (16.7%)	Meniscoplasty	410 (81.7%)
No	360 (71.7%)	Medial meniscus tear		Meniscoplasty with repair	16 (3.2%)
Yes	142 (28.3%)	No	484 (96.4%)	Total meniscectomy	76 (15.1%)
Involved knee joint		Yes	18 (3.6%)	Follow-up time (months)	75.4 (41.0 ~ 123.3; 33.7)
Left	252 (50.2%)	Site of knee cartilage lesion		Ikeuchi grade, preoperative	
Right	250 (49.8%)	No injury	358 (71.3%)	Poor	65 (12.9%)
Age of onset (years)	32.0 (3 ~ 80; 26.3)	Lateral compartment	100 (19.9%)	Fair	437 (87.1%)
Symptoms duration (months)	10.0 (0.05 ~ 246; 21.0)	Medial compartment	9 (1.8%)	Ikeuchi grade, postoperative follow-up	
Watanabe type		Patellofemoral joint	8 (1.6%)	Poor	13 (2.6%)
Complete	423 (84.3%)	Any two or more compartments	27 (5.4%)	Fair	44 (8.8%)
Incomplete	79 (15.7%)	Outerbridge grade		Good	105 (20.9%)
Site of DLM tear		No injury	358 (71.3%)	Excellent	340 (67.7%)

*BMI* body mass index, *K–L grade* Kellgren–Lawrence grade, *DLM* discoid lateral meniscus, *N* number, *M* median, *IQR* interquartile range

**Table 2:** Assignment of research factors for the clinical efficacy evaluation

Factors	Valuation
Sex	Female “0”; male “1”
BMI	N/A
Work intensity	Grade 0 “0”; grade 1 “1”; grade 2 “2”; grade 3 “3”; grade 4 “4”
Trauma history	No “0”; yes “1”
Involved knee joint	Left knee “0”; right knee “1”
Age of onset	N/A
Symptoms duration	N/A
Watanabe type	Type I (complete) “1”; type II (incomplete) “2”
Site of DLM tear	No “0”; anterior horn “1”; posterior horn “2”; body “3”; any two or more locations “4”
O'Connor type	No “0”; longitudinal (/bucket handle) tear “1”; horizontal tear “2”; oblique tear “3”; transverse (/radiation) tear “4”; variant tear (including flap, composite, degenerate meniscus tear) “5”
Medial meniscus tear	No “0”; yes “1”
Site of knee cartilage lesion	No “0”; lateral compartment “1”; medial compartment “2”; patellofemoral joint “3”; any two or more compartments “4”
Outerbridge grade	No “0”; level I “1”; level II “2”; level III “3”; level IV “4”
K–L grade	Level 0 “0”; level I “1”; level II “2”; level III “3”; level IV “4”
Surgical method	Meniscoplasty “1”; Meniscoplasty with repair “2”; Total meniscectomy “3”
Follow-up time	N/A
Ikeuchi classification	Excellent “3”; good “2”; fair and poor “1”

*BMI* body mass index, *K–L grade* Kellgren–Lawrence grade, *DLM* discoid lateral meniscus

The data were statistically analysed by SPSS 25.0. The normality test and homogeneity test of variance revealed that the measurement data does not satisfy the normal distribution and the homogeneity of variance. The measurement data and enumeration data were described by the median (M) and interquartile range (IQR), and the number of cases (percentage), respectively. The difference between the preoperative and postoperative functional assessments was analysed by the Wilcoxon rank-sum test. Univariate analysis: Kruskal–Wallis rank-sum test was used for measurement data among multiple groups; Mann–Whitney *U* test was applied to different analysis of two-category data, and Kruskal–Wallis rank-sum test to different analysis of rank data between multiple groups. Multivariate analysis: The model meets the parallelism by parallel test, and multivariate analysis was performed by ordinal logistic regression. *P* < 0.05 was considered statistically significant.

## Results

### General characteristics of the subjects

In this study, a sum of 502 patients was included, including 353 females (70.3%) and 149 males (29.7%); 252 (50.2%) left knees and 250 (49.8%) right knees. The median age of onset and symptoms duration were 32.0 years (range 3 ~ 80 years; IQR 26.3) and 10.0 months (range 0.05 ~ 246 months; IQR 21.0), respectively. The average follow-up was 75.4 months (range 41.0 ~ 123.3 months). There were 83 patients lost to follow up among 585 subjects, and the lost rate of follow up is 14.2%. According to Ikeuchi grade, preoperatively, 437 patients (87.1%) were rated as fair and 65 (12.9%) as poor; postoperatively, 340 patients (67.7%) were judged as excellent, 105 (20.9%) as good, 44 (8.8%) as fair and 13 (2.6%) as poor. Difference between preoperative and postoperative Ikeuchi grade was statistically significant (*P* < 0.001). (Table 3) The other features are shown in Table 1. In the follow-up after surgery, none of the patients required reoperation or had complications.

**Table 3:** Knee function Ikeuchi grade before and after the final follow-up

Knee function grade	Preoperative *N* (%)	Postoperative follow-up *N* (%)	*Z*	*P*
Ikeuchi grade			− 20.043	0.000
Poor	65 (12.9)	13 (2.6)		
Fair	437 (87.1)	44 (8.8)		
Good	0 (0.0)	105 (20.9)		
Excellent	0 (0.0)	340 (67.7)		

*N* number

*Statistically significant (*P* < 0.05)

### Univariate analysis of the 16 research factors and Ikeuchi grade

Factors such as sex, BMI, work intensity, trauma history, age of onset, symptoms duration, Watanabe type of DLM, combined medial meniscus tear, the site and Outerbridge grade of articular cartilage lesion, K–L grade and surgical method may be associated with Ikeuchi grade (*P* < 0.05). However, involved side of knee joint, site and O'Connor type of DLM tear, and follow-up time may not be correlated with Ikeuchi grade (*P* > 0.05) (Table 4).

**Table 4:** Univariate analysis of research factors and ikeuchi grade

Variable	Ikeuchi grade			*Z*/Kruskall–Wallis *χ*^2^	*P*
	Fair and poor	Good	Excellent		
Sex				− 3.615	0.000*
Female	45 (12.7)	87 (24.6)	221 (62.6)		
Male	12 (8.1)	18 (12.1)	119 (79.9)		
BMI	24.5 (4.3)	23.4(3.8)	21.0(4.0)	82.400	0.000*
Work intensity				47.059	0.000*
Grade 0	0 (0.0)	0 (0.0)	27 (100.0)		
Grade 1	16 (7.5)	28 (13.2)	168 (79.2)		
Grade 2	34 (14.2)	71 (29.6)	135 (56.3)		
Grade 3	7 (31.8)	6 (27.3)	9 (40.9)		
Grade 4	0 (0.0)	0 (0.0)	1 (100.0)		
Trauma history				− 2.610	0.009*
No	50 (13.9)	77 (21.4)	233 (64.7)		
Yes	7 (4.9)	28 (19.7)	107 (75.4)		
Involved knee joint				− 1.269	0.204
Left	25 (9.9)	50 (19.8)	177 (70.2)		
Right	32 (12.8)	55 (22.0)	163 (65.2)		
Age of onset	51.0 (22.5)	42.0(13.5)	24.0(23.0)	127.998	0.000*
Symptoms duration	12.0 (43.5)	12.0(33.0)	8.5(18.1)	15.050	0.001*
Watanabe type				− 2.110	0.035*
Complete	46 (10.9)	82 (19.4)	295 (69.7)		
Incomplete	11 (13.9)	23 (29.1)	45 (57.0)		
Site of DLM tear				2.066	0.724
No tearing	8 (21.1)	3 (7.9)	27 (71.1)		
Anterior horn	4(14.8)	8 (29.6)	15 (55.6)		
Posterior horn	6 (14.6)	8 (19.5)	27 (65.9)		
Body	16 (7.8)	49 (24.0)	139 (68.1)		
Two or more locations	23(12.0)	37(19.3)	132(68.8)		
O'Connor type				6.775	0.238
No tearing	8 (21.1)	3 (7.9)	27 (71.1)		
Longitudinal (/bucket handle) tear	16(9.4)	45 (26.3)	110 (64.3)		
Horizontal tear	12 (7.8)	28 (18.2)	114 (74.0)		
Oblique tear	2 (20.0)	1 (10.0)	7 (70.0)		
Transverse (/radial) tear	2 (4.4)	12 (26.7)	31 (68.9)		
Variant tear	17 (20.2)	16 (19.0)	51 (60.7)		
Medial meniscus tear				− 3.777	0.000*
No	46 (9.5)	105 (21.7)	333 (68.8)		
Yes	11 (61.1)	0 (0.0)	7 (38.9)		
Site of knee cartilage lesion				143.339	0.000*
No injury	11 (3.1)	56 (15.6)	291 (81.3)		
Lateral compartment	22 (22.0)	34 (34.0)	44 (44.0)		
Medial compartment	3 (33.3)	3 (33.3)	3 (33.3)		
Patellofemoral joint	5 (62.5)	2 (25.0)	1 (12.5)		
Any two or more compartments	16 (59.3)	10 (37.0)	1 (3.7)		
Outerbridge grade				145.995	0.000*
No injury	11 (3.1)	56 (15.6)	291 (81.3)		
Level I	0 (0.0)	10 (34.5)	19 (65.5)		
Level II	15 (28.8)	20 (38.5)	17 (32.7)		
Level III	8 (34.8)	11 (47.8)	4 (17.4)		
Level IV	23 (57.5)	8 (20.0)	9 (22.5)		
K–L grade				155.520	0.000*
Level 0	10 (2.7)	64 (17.1)	301 (80.3)		
Level I	10 (17.9)	17 (30.4)	29 (51.8)		
Level II	19 (43.2)	20 (45.5)	5 (11.4)		
Level III	14 (60.9)	4 (17.4)	5 (21.7)		
Level IV	4 (100.0)	0 (0.0)	0 (0.0)		
Follow-up time	71.0 (33.2)	79.9 (40.7)	74.9 (31.4)	4.722	0.094
Surgical method				85.971	0.000*
Meniscoplasty	23 (5.6)	76 (18.5)	311 (75.9)		
Meniscoplasty with repair	2 (12.5)	6 (37.5)	8 (50.0)		
Total meniscectomy	32 (42.1)	23 (30.3)	21 (27.6)		

*BMI* body mass index, *K–L grade* Kellgren–Lawrence grade

*Statistically significant (*P* < 0.05)

### Multivariate analysis of the 16 research factors and Ikeuchi grade

Female was an adverse factor for obtaining the higher Ikeuchi grade [*P* = 0.009, odds ratio (OR) 0.45, 95% confidence interval (CI) 0.255–0.822]. Outerbridge grade of articular cartilage lesion (*P* = 0.018, OR 0.638, 95% CI 0.438–0.927) was negatively correlated with Ikeuchi grade. BMI and work intensity were inversely correlated with Ikeuchi grade (*P* = 0.001, OR 0.875, 95% CI 0.808–0.948 and *P* = 0.020, OR 0.611, 95% CI 0.404–0.926, respectively). Age of onset (*P* < 0.001, OR 0.956, 95% CI 0.933–0.979) and symptoms duration (*P* < 0.001, OR 0.988, 95% CI 0.983–0.994) were negatively correlated with Ikeuchi grade. Besides, compared to total meniscectomy, meniscoplasty with repair was an unfavourable factor for getting good Ikeuchi grade (*P* = 0.044, OR 0.245, 95% CI 0.062–0.963). Nevertheless, no significant difference was found between total meniscectomy and meniscoplasty. In addition, K–L grade, history of trauma, Watanabe type of DLM, combined medial meniscus tear and site of articular cartilage lesion did not affect Ikeuchi grade (Table 5).

**Table 5:** Multivariate analysis of research factors and Ikeuchi grade

Variable	*B*	S.E	Wald	*P*	OR	95% CI	
						Lower	Upper
BMI	− 0.133	0.041	10.610	0.001*	0.875	0.808	0.948
Work intensity	− 0.492	0.212	5.400	0.020*	0.611	0.404	0.926
Age of onset	− 0.045	0.012	13.391	0.000*	0.956	0.933	0.979
Symptoms duration	− 0.012	0.003	18.767	0.000*	0.988	0.983	0.994
Outerbridge grade	− 0.450	0.191	5.557	0.018*	0.638	0.438	0.927
K–L grade	− 0.239	0.193	1.540	0.215	0.787	0.540	1.149
Sex							
Female	− 0.780	0.298	6.843	0.009*	0.458	0.255	0.822
Male^a^	–	–	–	–	1.000	–	–
Trauma history							
No	− 0.310	0.277	1.249	0.264	0.733	0.426	1.262
Yes^a^	–	–	–	–	1.000	–	–
Watanabe type							
Complete	0.097	0.317	0.093	0.760	1.102	0.592	2.048
Incomplete^a^	–	–	–	–	1.000	–	–
Medial meniscus tear							
No	0.619	0.606	1.043	0.307	1.857	0.567	6.086
Yes^a^	–	–	–	–	1.000	–	–
Site of knee cartilage lesion							
No injury	0.354	0.796	0.198	0.657	1.425	0.299	6.780
Lateral compartment	0.039	0.554	0.005	0.943	1.040	0.351	3.080
Medial compartment	0.129	0.851	0.023	0.879	1.138	0.215	6.032
Patellofemoral joint	− 0.434	0.923	0.221	0.638	0.648	0.106	3.959
Any two or more compartments^a^	–	–	–	–	1.000	–	–
Surgical method							
Meniscoplasty	0.325	0.351	0.856	0.355	1.384	0.696	2.751
Meniscoplasty with repair	− 1.406	0.698	4.056	0.044*	0.245	0.062	0.963
Total meniscectomy^a^	–	–	–	–	1.000	–	–

*BMI* body mass index, *K–L grade* Kellgren–Lawrence grade, *OR* odds ratio, *CI* confidence interval

*Statistically significant (*P* < 0.05)

^a^Represent Reference group

## Discussion

In this study, we found that sex, BMI, work intensity, age of onset, duration of symptoms, the severity of cartilage lesion and surgical mode are correlated with postoperative outcomes of symptomatic DLM, while K–L grade, history of trauma, Watanabe type of DLM, combined medial meniscus tear and site of articular cartilage lesion did not correlate with Ikeuchi grade.

Female is a risk factor for many orthopaedic diseases, but its correlation with postoperative efficacy of symptomatic DLM is unclear. Chen [17], Kose [9] and Higuchi [18] all found that sex exerts no significant effect on the postoperative outcomes by analysing, respectively, 39 patients, 48 patients and 67 patients with DLM. However, Ahn et al. [19], by evaluating 260 DLM patients, believed that sex could affect postoperative outcomes and that male is a protective factor. This study found that sex can influence Ikeuchi grade (*P* < 0.05) and that female is a risk factor. Studies have shown that the knee articular cartilage volume of women is significantly smaller than that of men and that the female *Q* angle is greater than that of male, leading to increased patellofemoral joint pressure; thus, women are more prone to suffer cartilage lesions and osteoarthritis than men [8, 20–22]. This may be the reason for poor postoperative clinical outcomes in women.

Overweight negatively impacts musculoskeletal health [23]. Fu et al. [8] observed that patients with BMIs > 23.0 kg/m^2^ are more likely to suffer from articular cartilage lesions than patients with low BMIs. However, Ahn et al. [24] believed that BMI is not a risk factor for radiographic progression of postoperative osteoarthritis of DLM tear. This study found that BMI is negatively correlated with Ikeuchi grade. The higher the BMI, the lower the Ikeuchi grade is. Reportedly, obesity can lead to meniscus compression, and pathological change and loss of articular cartilage, which ultimately results in knee osteoarthritis [25–27]. Besides, we detected that work intensity is inversely related to Ikeuchi grade, which may be because knee joint activity, load-bearing capacity and cartilage lesions are positively correlated with work intensity.

Symptoms of DLM can be onset at any age. Younger age of onset may be related to a lower risk of postoperative chondromalacia and a better outcome because of the earlier diagnosis and treatment [7, 28]. Chen [17] and Lee [6] believed that a longer duration of symptoms is associated with a worse efficacy of arthroscopic surgery. Most studies have shown that a longer duration of symptoms, especially > 6 months, may be more prone to make lateral cartilage and residual meniscus injured and degenerated [6–8, 24, 29]. This study found that age of onset (*P* < 0.001, OR 0.956, 95% CI 0.933–0.979) and duration of symptoms (*P* < 0.001, OR 0.988, 95% CI 0.983–0.994) are negatively correlated with the postoperative outcomes. Therefore, earlier diagnosis and treatment of symptomatic DLM would lead to a better postoperative outcome.

Similar to Kose et al. [9], for postoperative outcomes of DLM patients, we found that Outerbridge grade of the cartilage lesion was negatively correlated with Ikeuchi grade, and that site of the cartilage lesion did not affect Ikeuchi grade. Outerbridge grade can directly reflect the severity of the knee cartilage lesions. Articular cartilage lesions are rarely self-healing. Although the clinical manifestations of an articular cartilage lesion may not be obvious in the short term, most patients will eventually deteriorate to irreversible knee osteoarthritis, thus drastically affecting knee function [30, 31]. In addition, even if K–L grade is associated with the Outerbridge grade, we did not find the significant effect of K–L grade on the postoperative outcome. A higher K–L grade is associated with a more severe cartilage lesion in MRI [32]. However, some studies have found that X-rays are less sensitive in detecting cartilage lesions and articular cartilage loss as early narrowing in joint space is not secondary to articular cartilage thinning but is secondary to meniscal compression [33, 34].

Treating symptomatic DLM by arthroscopic surgery, meniscoplasty is the first choice [3, 24, 28, 35], and meniscoplasty with repair and total meniscectomy are also options. In terms of the postoperative outcomes of these three surgical methods, the opinion is inconsistent. Wong et al. [28] concluded that there was no significant difference in the postoperative outcomes among these three surgical methods. Some studies have found no difference between meniscoplasty and total meniscectomy as to short-term clinical outcomes [36–38], but the clinical efficacy of meniscoplasty is better than that of total meniscectomy in the long-term follow-up [3, 24, 38–42]. Other studies have shown that the long-term outcomes of meniscoplasty with or without repair are satisfactory, without statistical difference [9, 38, 39, 41, 43, 44]. Moreover, Perkins et al. [45] founded that, for meniscocapsular tears, meniscoplasty with repair is related to low rates of revision surgery and good intermediate-term outcomes. Geffroy [46] observed that the results of meniscoplasty with repair in children with DLM are very satisfactory in general, whatever the type or site of the lesion. Conversely, Lee et al. [6] believe that residual discoid meniscus tissue is prone to degeneration and re-injury due to abnormally fibrous structure, which may lead to adverse clinical effects. Besides, considering the high cost and uncertain effective of the repair, Smuin et al. [38] do not recommend the repair of the abnormal anatomy in a torn DLM. In the present study, compared with the postoperative efficacy of total meniscectomy, meniscoplasty seems to be no different, but meniscoplasty with repair seems worse (*P* = 0.044, OR 0.245). This result, however, seems to be inconsistent with the results of statistical description that percentage of “excellent" and "good" in meniscoplasty with repair group is higher than that in total meniscectomy group (87.5% vs 57.9%), which may be attributed to the characteristics of multivariate analysis model and small sample size. Compared with statistical description, the multivariate analysis model can independently reflect the effect of the surgical method on Ikeuchi grade by controlling the influence of other factors. Besides, the small sample size in meniscoplasty with repair and total meniscectomy may reduce the power of the test to some extent. Therefore, the influence of surgical methods on the postoperative efficacy of DLM needs to be further verified by extending the follow-up time, expanding the sample size, and increasing the postoperative imaging evaluation.

So far, there are few studies on the effects of the site of DLM tear on postoperative outcomes. Hede et al. [47] found that when the amount of meniscal tissue removed is less than 30%, the site of the lesion does not affect the postoperative outcome; but more than 30%, the postoperative outcome of tears in anterior and posterior horn is worse than that of meniscal body tears. Moreover, studies have shown that tear type does not affect the postoperative outcome [8, 9, 19]. However, Chen [17] and Badlani [48] considered that radial tears in the discoid meniscus led to poor postoperative outcome. Ahn et al. [26] found that horizontal tears in the discoid meniscus were an important risk factor for the radiological progression of postoperative osteoarthritis with K–L grade 3/4. In our study, the site and O'Connor type of the DLM tear do not affect the postoperative outcomes, which may be because patients with DLM tears often diagnosed and treated early for their clinical symptoms. Besides, consistent with the result of Lee [6] and Kose [9], this study also showed that the Watanabe type of DLM is not an influencing factor for the postoperative outcomes, which probably because the incidence of a cartilage lesion is not correlated with the DLM type [8, 29]. Although a longer follow-up period is believed to be associated with a bad knee function score [6, 40]. our study did not demonstrate that the final follow-up time (75.4, 41 ~ 123 months) is an influencing factor for the postoperative efficacy, which may be due to the small number of patients with follow-up periods over 120 months.

We acknowledge that there are some limitations to our study. First, the postoperative efficacy evaluation in this study did not assess the objective imaging changes at the final follow-up and instead only evaluated the subjective functional parameters. Second, the lost rate of follow-up is 14.2%, which may be a potential source of bias. Third, comparatively small sample size, and multi-categorized factors and Ikeuchi grade result in a few patients in some categories, which may lower the power of the test. Finally, this study is a retrospective multivariate analysis, and the conclusions need to be further confirmed by prospective studies.

## Conclusion

With the increase of BMI, work intensity, age of onset, duration of symptoms, and the severity of cartilage lesion, the postoperative results become worse. Moreover, female and meniscoplasty with repair are risk factors for the postoperative outcomes.

## Supplementary Information

Below is the link to the electronic supplementary material.

Supplementary file1 (DOCX 149 KB)

Supplementary file2 (XLSX 62 KB)

## Data Availability

The patient's personal information and imaging data obtained at following-up were stored on the disc. The data are available from the corresponding author upon request. GC should be contacted with requests for data and materials.
