# Supplementary material for: Multivariate ordered logistic regression analysis of the postoperative effect of symptomatic discoid lateral meniscus
Source: Arch Orthop Trauma Surg. 2021 Feb 22;141(11):1935–44. doi: 10.1007/s00402-021-03821-3 (PMC8497286; doi:10.1007/s00402-021-03821-3)
Supplement: Supplementary file 1 — Supplementary file1 (DOCX 149 KB) [file 402_2021_3821_MOESM1_ESM.docx]

Schedule 1 REFA classification of workload Grade Work intensity

| Grade | Work intensity | Example |
| --- | --- | --- |
| 0 | Work without special physical strain | Work without load like, for example, desk work |
| 1 | Work with small physical strain | Handling light work pieces; lengthy standing or walking around |
| 2 | Work with moderate physical strain | Handling of 1–3 kg control device; carrying loads of 10–15 kg; climbing stairs or ladders without load |
| 3 | Work with hard physical strain | Carrying loads of 20–30 kg, shovelling, digging, chipping, climbing stairs or ladders with moderate load, moderate work in tense work posture |
| 4 | Work with most heavily physical strain | Carrying loads of more than 50 kg, climbing with heavy load, hard work in tense work posture |

Schedule 2 Watanabe classification of discoid lateral meniscus

| Type | Description |
| --- | --- |
| I | Complete |
| II | Incomplete |
| III | Wrisberg ligament variant, lack of posterior meniscotibial attachment to the tibia |

Schedule 3 Kellgren-Lawrence Classification criteria of knee Osteoarthritis

Grade 0 normal

Grade 1 Doubtful narrowing of joint space and possible osteophytic lipping

Grade 2 Definite osteophytes and possible narrowing of joint space

Grade 3 Moderate multiple osteophytes, definite narrowing of joint space and some sclerosis and possible deformity of bone ends

Grade 4 Large osteophytes, marked narrowing of joint space, severe sclerosis and definite deformity of bone ends

Schedule 4 Meniscus tear O'Connor classification

| Meniscus tear type | Schematic diagram |
| --- | --- |
| longitudinal (/bucket handle) tear | 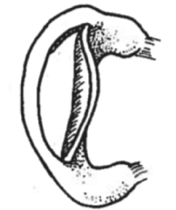 |
| horizontal tear | 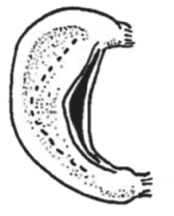 |
| oblique tear | 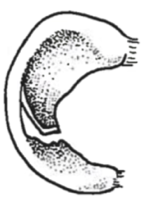 |
| transverse (/radiation) tear | 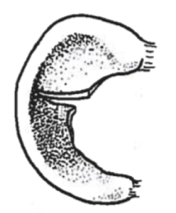 |
| variant tear (including flap,  composite, degenerate meniscus tear) | 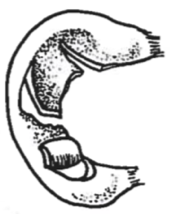 |

Schedule 5. Outerbridge classification

| Grade | Pathology |
| --- | --- |
| I | Softening and swelling of articular cartilage |
| II | Fragmentation and fissuring of articular cartilage affecting an area of less than 0.5 inches |
| III | Fragmentation and fissuring of articular cartilage affecting an area greater than 0.5 inches |
| IV | Cartilage erosion to bone |

Schedule 6 Grading Scale of Ikeuchi

| Grade | Description |
| --- | --- |
| excellent | no limitation of motion, click, noise or pain |
| good | occasional slight pain on motion, but no motion symptoms |
| fair | slight pain and in addition, a click or noise with motion without limitation of motion |
| poor | pain at rest as well as with motion and limitation of motion |
